# 12 Tips for Including Disability Awareness within Undergraduate Medical Education Curricula

**DOI:** 10.12688/mep.20329.1

**Published:** 2024-05-14

**Authors:** Tanvee Sinha, William S. Brooks, Ashley Parish

**Affiliations:** 1 Marnix E. Heersink School of Medicine, The University of Alabama at Birmingham, Birmingham, Alabama, 35294, USA; 2 Department of Cell, Developmental & Integrative Biology, The University of Alabama at Birmingham, Birmingham, Alabama, 35294, USA; 3 Department of Physical Therapy, The University of Alabama at Birmingham, Birmingham, Alabama, 35294, USA

**Keywords:** Disability awareness, Undergraduate medical education, Americans with Disabilities Act, Interprofessional education

## Abstract

Persons with disabilities (PWD) make up the largest minority group in the U.S. and experience significant health care disparities. Effective training for physicians who will inevitably encounter these patients is warranted; however, as low as 23% of American medical schools have a disability curriculum, which has resulted in a sense of unpreparedness and discomfort among physicians when caring for PWD. The goal of disability training is to provide the proper knowledge and skills to address the unique needs of PWD, mitigate health disparities, and help shape more compassionate and informed physicians. This article presents 12 tips to incorporate disability training into undergraduate medical education. These tips emphasize the inclusion of PWD in all stages of the curriculum, interprofessional education, experiential learning, and exposure to a range of disability types. By leveraging these tips, educators will be able to create effective learning opportunities and improve the future healthcare of PWD.

## Introduction

With an estimated population of approximately 50 million, persons with disabilities (PWD) comprise the largest minority group in the U.S. and contribute significantly to the diversity of U.S. residents ( Leppert & Schaeffer, 2023; Okoro *et al.*, 2018). PWD face significant health care disparities due in part to their complex medical needs and barriers faced when accessing medical care. As a result, chronic diseases such as obesity, diabetes, and heart disease are more prevalent within this vulnerable population ( CDC, 2020). Physicians note that a variety of barriers limit their ability to provide effective care for these individuals, including knowledge gaps, challenges with communication, and a lack of accessible facilities ( McMillan *et al.*, 2016). Fewer than half of physicians surveyed in one study reported comfort when providing care for this population ( Holder *et al.*, 2009). Early and frequent exposure of medical trainees to PWD, their unique health care needs, and barriers to effective care is one strategy to address these health disparities and improve medical care for these individuals. 

The following 12 tips are provided for medical educators wishing to incorporate disability awareness into the medical training programs at their own institutions. These tips are based upon the authors’ experiences with disability awareness education in both undergraduate medical education (UME) and graduate medical education (GME) ( Parish *et al.*, 2024; Sinha *et al.*, 2024). These tips have been developed within the theoretical framework of intergroup contact theory (ICT), which stresses the importance of direct contact between minority and majority groups to break down prejudices and foster collaboration and shared understanding ( Pettigrew, 1998; Pettigrew & Tropp, 2006). A key tenant of any disability awareness program is the inclusion of PWD into every phase of the process, from development through implementation and assessment.

### Tip 1 – Identify gaps in knowledge and use data to form learning objectives and drive curricular design

 Medical students enter training from a wide array of backgrounds and experiences, and there is no “one-size-fits-all" model for providing disability awareness education. To ensure success, one needs to understand the knowledge base of students at their institution well. A needs assessment survey and/or set of focus groups with students can identify baseline knowledge and experiences around disability, which can be used to tailor a disability awareness curriculum to meet the unique needs of each medical school. Learning objectives should aim to address gaps in learner knowledge and align with programmatic learning goals. Curricular design should align with the overall curricular approach and culture of your medical school to establish continuity of instruction and further contribute to success. For example, if problem-based learning (PBL) is a core curricular element of your medical school, then including patients who have disabilities in one or more PBL cases would be an appropriate strategy. If students routinely engage in service learning, then partnering with community centers that provide services to PWD would be a great option.

### Tip 2 – Embed PWD into all phases of the curriculum

Coined during the disability civil rights movement, the slogan “nothing about us without us” refers to the idea that no policy should be decided without involvement of members of the affected group ( Chu *et al.*, 2016). Multiple factors contribute to lower quality health care delivery to PWD, which contributes to further health care disparities and poorer health outcomes; these factors would be realistically modifiable with input from PWD ( Sharby *et al.*, 2015). PWD must be included in all aspects of disability awareness curricula, including the development (design and content), implementation, and assessment phases. Those who possess lived experiences and voices are most capable of making a longitudinal effect on future medical practice. Failure to engage with PWD can lead to the propagation of ableist ideas and distortion of the most important facets of disability and healthcare. Developing partnerships with local rehabilitation clinics and community centers are great ways to create professional contacts to foster a collaborative approach to disability awareness and recruit PWD to participate as educators. 

### Tip 3 – Utilize diverse clinicians with experience working with PWD

The Centers for Disease Control and Prevention classifies disability into six types: mobility, cognition, independent living, hearing, vision, and self-care ( CDC, 2020). Some medical and health professions, such as physical therapy, occupational therapy, speech-language pathology and physiatry, have specific roles in caring for PWD who have one or more of these disability types. Because of their prevalent roles working with this population, disability awareness is often an accreditation requirement and embedded within their training. For example, Doctor of Physical Therapy (DPT) programs integrate disability awareness, distinctively geared toward mobility-based disability, throughout their curricula ( Roush & Sharby, 2011). Therefore, in addition to PWD, it is helpful to integrate healthcare professionals who have experience working with this population into the disability awareness programs. This is particularly important as PWD often require a team-based approach to care that includes a variety of health professions ( Reis *et al.*, 2004). Inclusion of clinicians who possess these experiences can help ensure that key considerations and clinical pearls are embedded within the curricula.

### Tip 4 – Include Interprofessional Education (IPE) elements

Emphasizing an interprofessional approach when implementing disability awareness curricula is important, because caring for PWD often requires communication and planning as a healthcare team ( Iezzoni, 2006; Reis *et al.*, 2004). IPE has been shown as beneficial to learning, skill development, and creating a plan of care ( McCave *et al.*, 2019; Visser *et al.*, 2019). It is also important for future physicians to understand the role of other healthcare professionals in treating PWD as it can allow for better patient-client management and referral for those who may have unmet needs. Since many medical schools have associated health professions programs, IPE activities can involve students from physical therapy, occupational therapy, speech-language pathology, nursing, and physician associate programs to name a few. Disability awareness activities provide a robust and practical setting to meet accreditation requirements around IPE.

### Tip 5 – Incorporate diversity and inclusion into the curriculum

Disability is neither a single diagnosis nor a single human characteristic. It is a collection of physical or mental impairments that limits life activities, and it can result from myriad illnesses and injuries. Furthermore, people with disabilities are just as diverse as those who do not have a disability. Educational programs that promote disability awareness should strive to be as inclusive as possible and represent the full diversity of this population. One way to do this is to include a breadth of disability types in your instruction and learning experiences:

- Mobility – You could incorporate wheelchair users (manual and powerchair) and have students self-propel themselves in wheelchairs around your educational building.
- Cognition – You could allow students to volunteer at community centers or other sites that provide services to individuals with intellectual and developmental disabilities.
- Vision – You could incorporate a patient with a vision impairment into a PBL or case-based learning session or allow students to volunteer at training sites for service animals.
- Hearing – You could provide a medical sign language class or allow students to practice working with an interpreter to communicate with guests who have hearing impairments.

Be sure to engage a diverse group of PWD in the planning and implementation of the program, ensuring diversity of age, sex, gender, race, ethnicity, disability type, disability etiology (congenital vs. acquired), occupation, etc.

### Tip 6 – Include a variety of pedagogies, emphasizing experiential learning

 Successful promotion of disability awareness requires more than didactics alone can provide. Utilize a variety of instructional methods, pairing your pedagogy with intended outcomes. Lectures, readings, or videos are great for communicating about the Americans with Disabilities Act (ADA) and providing tips for communication etiquette. Modifying existing clinical cases to include a disability element is an easy way to incorporate awareness without adding new educational sessions to curricula that may already be crowded. Whenever possible, experiential learning, which includes direct interaction between students and PWD, should be considered as a focal element of disability curricula. Service learning and community outreach activities allow students to see and interact with PWD in non-clinical settings. Bringing PWD onto campus for small group discussions is another way to create valuable learning experiences.

### Tip 7 – Ensure access to appropriate equipment and facilities

PWD often utilize assistive and adaptive equipment to help promote independence. As physicians are often the prescribers of equipment, it is imperative to have familiarity with types of equipment and spaces in which equipment is utilized. For example, a commonly prescribed piece of equipment for both short- and long-term mobility impairments is a wheelchair. Having access to various types of wheelchairs during interactive disability awareness learning activities can allow for discussion of costs, proper fit, and parts of wheelchairs that may be needed based on diagnosis. Similarly, gait belts and sliding boards are useful to demonstrate how a PWD may transfer to and from an examination table. For language disabilities, communication devices could be presented. Equipment integrated into the disability awareness curriculum should be diverse and based upon the type of disability being studied to allow for holistic exposure and understanding of the compatible devices that PWD may use.

### Tip 8 – Introduce students to the Americans with Disabilities Act (ADA)

 The Americans with Disabilities Act (ADA) is the civil rights law that was enacted in 1990 to prohibit discrimination against individuals with disabilities, ensuring that this population has the same rights and opportunities as those without disabilities. Title III of the ADA sets the minimum standards for accessibility of public and privately-owned facilities, directs businesses to make reasonable accommodations when serving people with disabilities, and requires appropriate measures be taken to effectively communicate with individuals who have speech, hearing and vision impairments. Introducing medical students to the basic tenants of the ADA is an essential component of any disability awareness curriculum as it forms the foundation for government-mandated accommodation and accessibility considerations. This can be done through short videos or reading assignments. Other approaches that employ more active methods of instruction include one-on-one and small group conversations about accessibility with individuals who have disabilities and placing students into a variety of ADA compliant and non-compliant facilities. Our approach to teaching ADA tenants includes both lecture-based content and experiential learning.

### Tip 9 – Simulate first-hand experiences by placing learners “into the shoes” of PWD

It is important to note that although experiential learning may allow a student to simulate what it is like to have a disability, it is not the same as living with a disability. For our approach, PWD help to create and lead experiential learning activities. This allows medical students to have a more meaningful learning opportunity versus inducing a sympathy-based response for individuals who live with disabilities. For our curriculum, physical therapy students teach medical students how to self-propel a wheelchair. Medical students then self-propel wheelchairs through their educational building alongside individuals who use wheelchairs for mobility. The wheelchair users guide students to experience the ADA compliant and non-compliant aspects of buildings, sidewalks, and parking during this process. The wheelchair users then have an open dialogue with students to discuss the long-term effects of using a wheelchair for mobility, share their experiences, and answer questions. These meaningful learning contexts improve student engagement, understanding, and retention of the material all while promoting deeper awareness of the emotional, social, and physical aspects of living with a disability ( Laurillard, 2012). Immersing students in these situations helps break down stereotypes, while building a sense of responsibility to improve the lack of inclusivity, the “non-disability-friendly” spaces, and increase patience in their future clinical practice.

### Tip 10 – Create opportunities for open dialog between learners and PWD

Creating opportunities for open dialogue between students and PWD who serve as the facilitators of learning experiences is crucial to fostering a more effective learning environment. These interactions help dispel misconceptions, bridge gaps between learners and facilitators, and foster more encouraging and accepting clinical practice. PWD are underrepresented as educators, but they may reflect images in the mirror for many students and help guide learners in understanding everyday challenges and triumphs for PWD ( Anderson *et al.*, 1998). Open channels of communication in the learning environment help build collaborative relationships where facilitators and learners can discuss shared perspective and various views surrounding them as well as uncomfortable but necessary dilemmas ( Ta *et al.*, 2023). Through open discussions, everyone’s unique abilities and perspectives are acknowledged and integrated to better enhance learning.

### Tip 11 – Embed reflective elements into the activities

Adding reflective elements, such as small group debriefs and reflection essays, into a disability curriculum can enhance an already dynamic and inclusive learning environment for learners and facilitators alike. For learners, research has shown that when students combine their learning with reflection, they are able to effectively understand and analyze problems that they could not otherwise ( Eyler & Giles, 1999). When students are encouraged to recognize their strengths and weaknesses and assess their learning through self-reflection, they are more likely to adopt those processes going forward ( Silverman & Cassaza, 2000). For facilitators, reflection provides structure to their teaching and encourages them to take responsibility for themselves. By understanding what is effective and what could be improved upon, reflection helps educators create more inclusive curricula with greater relevance and direction for their learners ( Daudelin, 1996). Overall, incorporating reflective elements in a disability-based curriculum can enhance the experience for learners and stress continuity while also contributing to the professional growth of PWD as educators, creating a more enriching ecosystem.

### Tip 12 – Create longitudinal experiences

Currently, over half of U.S. medical schools lack a disability awareness program ( Holder *et al.*, 2009). For those that do, most incorporate training within the pre-clinical years but fail to provide experiences in the clinical phase of training, which limits opportunities for application in clinical practice. Both the lack of education and continuity adds to the negative bias and discomfort that many medical students and physicians feel when treating PWD; this only results in worse healthcare and medical attention for PWD ( Sharby *et al.*, 2015). The ineffective nature of continued medical education contributes to the discrepancy between evidence/experience-based knowledge and fair practice ( Marinopoulos *et al.*, 2007). Thus, it is vital that schools not only incorporate disability training early in medical training but thread these experiences longitudinally throughout training. Ensuring students encounter PWD during clerkships, offering advanced electives in the care of PWD, and providing opportunities for team-based healthcare with physical or occupational therapists are ways in which disability awareness education can be brought into the clinical phase of training. Residency programs should also consider how to incorporate these essential skills into their curricula.

## Conclusion

The goal of these 12 tips is to provide medical educators with the necessary tools to promote students’ awareness of unique considerations when providing care for PWD and foster a platform for PWD to function as key participants in the learning environment. It is crucial that medical schools prepare the next generation of physicians to provide effective healthcare for this population through diverse teaching and learning methods, interprofessional collaboration, and a focus on reflection and continuity. By embracing these tips, the teaching and learning process can become enriching for all stakeholders and cultivate trainees who will be compassionate and confident providers within their future roles.

## Ethics and consent

Ethical approval and Consent were not required.

## Data Availability

No data are associated with this article.

## References

[ref-1] Anderson RJ Keller CE Karp JM : Enhancing diversity: educators with disabilities.Washington, D.C., Gallaudet University Press,1998. 10.2307/j.ctv2rcnggb

[ref-2] CDC: Disability and health overview. 2020; Retrieved 18 March 2024. Reference Source

[ref-3] Chu LF Utengen A Kadry B : "Nothing about us without us"-patient partnership in medical conferences. *BMJ.* 2016;354: i3883. 10.1136/bmj.i3883 27628427

[ref-4] Daudelin MW : Learning from experience through reflection. *Organ Dyn.* 1996;24(3):36–48. 10.1016/S0090-2616(96)90004-2

[ref-5] Eyler J Giles D : Where's the learning in service learning.San Francisco, CA, Jossey-Bass Publishers,1999.

[ref-6] Holder M Waldman HB Hood H : Preparing health professionals to provide care to individuals with disabilities. *Int J Oral Sci.* 2009;1(2):66–71. 10.4248/ijos.09022 20687298 PMC3735794

[ref-7] Iezzoni LI : Going beyond disease to address disability. *N Engl J Med.* 2006;355(10):976–979. 10.1056/NEJMp068093 16957143

[ref-8] Laurillard D : Teaching as a design science: building pedagogical patterns for learning and technology.London, U.K., Routledge,2012. Reference Source

[ref-9] Leppert R Schaeffer K : 8 facts about Americans with disabilities. 2023; Retrieved 18 March 2024. Reference Source

[ref-10] Marinopoulos SS Dorman T Ratanawongsa N : Effectiveness of continuing medical education. *Evid Rep Technol Assess (Full Rep).* 2007; (149):1–69. 17764217 PMC4781050

[ref-11] McCave EL Aptaker D Hartmann KD : Promoting affirmative transgender health care practice within hospitals: an IPE standardized patient simulation for graduate health care learners. *MedEdPORTAL.* 2019;15: 10861. 10.15766/mep_2374-8265.10861 32051844 PMC7010321

[ref-12] McMillan C Lee J Milligan J : Physician perspectives on care of individuals with severe mobility impairments in primary care in Southwestern Ontario, Canada. *Health Soc Care Community.* 2016;24(4):463–472. 10.1111/hsc.12228 25809600

[ref-13] Okoro CA Hollis ND Cyrus AC : Prevalence of disabilities and health care access by disability status and type among adults - United States, 2016. *MMWR Morb Mortal Wkly Rep.* 2018;67(32):882–887. 10.15585/mmwr.mm6732a3 30114005 PMC6095650

[ref-14] Parish A Carver C Lein DH Jr : Come Roll with Me: an interprofessional experience to promote disability awareness. *Teach Learn Med.* 2024;36(2):183–197. 10.1080/10401334.2022.2148107 36426664

[ref-15] Pettigrew TF : Intergroup contact theory. *Annu Rev Psychol.* 1998;49:65–85. 10.1146/annurev.psych.49.1.65 15012467

[ref-16] Pettigrew TF Tropp LR : A meta-analytic test of intergroup contact theory. *J Pers Soc Psychol.* 2006;90(5):751–783. 10.1037/0022-3514.90.5.751 16737372

[ref-17] Reis JP Breslin ML Iezzoni LI : It takes more than ramps to solve the crisis of healthcare for people with disabilities.Chicago, IL, Rehabilitation Institute of Chicago,2004. Reference Source

[ref-18] Roush SE Sharby N : Disability reconsidered: the paradox of physical therapy. *Phys Ther.* 2011;91(12):1715–1727. 10.2522/ptj.20100389 22003161

[ref-19] Sharby N Martire K Iversen MD : Decreasing health disparities for people with disabilities through improved communication strategies and awareness. *Int J Environ Res Public Health.* 2015;12(3):3301–3316. 10.3390/ijerph120303301 25809511 PMC4377965

[ref-20] Silverman S Cassaza M : Learning and development: making connections to enhance teaching.San Francisco, CA, Jossey-Bass Publishers,2000. Reference Source

[ref-21] Sinha T Parish A Lein DH : Integration of disability awareness improves medical students’ attitudes toward people with disabilities. *Med Sci Educ.* 2024. 10.1007/s40670-024-02004-0 PMC11180063 38887426

[ref-22] Ta T Geron T Levinson M : "The power of open dialogue": using normative case studies to facilitate ethical dilemmas discussions among school teachers. *Teaching and Teacher Education.* 2023;132: 104237. 10.1016/j.tate.2023.104237

[ref-23] Visser CLF Kusurkar RA Croiset G : Students' motivation for interprofessional collaboration after their experience on an IPE ward: a qualitative analysis framed by self-determination theory. *Med Teach.* 2019;41(1):44–52. 10.1080/0142159X.2018.1436759 29490575

