# 12 Tips for Including Disability Awareness within Undergraduate Medical Education Curricula

**DOI:** 10.12688/mep.20329.3

**Published:** 2024-12-10

**Authors:** Tanvee Sinha, Caroline N. Harada, William S. Brooks, Ashley Parish

**Affiliations:** 1 Marnix E. Heersink School of Medicine, The University of Alabama at Birmingham, Birmingham, Alabama, 35294, USA; 2 Department of Medical Education, The University of Alabama at Birmingham, Birmingham, Alabama, 35294, USA; 3 Department of Cell, Developmental & Integrative Biology, The University of Alabama at Birmingham, Birmingham, Alabama, 35294, USA; 4 Department of Physical Therapy, The University of Alabama at Birmingham, Birmingham, Alabama, 35294, USA

**Keywords:** Disability awareness, Undergraduate medical education, Americans with Disabilities Act, Interprofessional education

## Abstract

Disability is extremely common, and there is a need for high quality medical school curricula on working with persons with disabilities. The goal of disability training is to provide the proper knowledge and skills to address the unique needs of persons with disability (PWD), mitigate health disparities, and help shape more compassionate and informed physicians. This article presents 12 tips to incorporate disability training into undergraduate medical education. These tips emphasize the inclusion of PWD in all stages of the curriculum, interprofessional education, experiential learning, and exposure to a range of disability types. By leveraging these tips, educators will be able to create effective learning opportunities and improve the future healthcare of PWD.

## Introduction

With an estimated population of approximately 70 million, persons with disabilities (PWD) comprise the largest minority group in the U.S. and contribute significantly to the diversity of U.S. residents ( CDC, 2024; Leppert & Schaeffer, 2023; Okoro *et al.*, 2018). PWD face significant health care disparities due in part to their complex medical needs and barriers faced when accessing healthcare. As a result, chronic diseases such as obesity, diabetes, and heart disease are more prevalent within this vulnerable population ( CDC, 2024). Physicians note that a variety of barriers limit their ability to provide effective care for these individuals, including knowledge gaps, challenges with communication, and a lack of accessible facilities ( McMillan *et al.*, 2016). Approximately half of American medical schools have a disability curriculum, which may be why fewer than half of physicians surveyed in one study reported comfort when providing care for this population ( Holder *et al.*, 2009; Seidel & Crowe, 2017). Early and frequent exposure of medical trainees to PWD, their unique health care needs, and barriers to effective care is one strategy to address these health disparities and improve healthcare for these individuals. It must be noted that instruction regarding the pathophysiology, diagnosis and management of disabling conditions is not equivalent to instruction regarding disability. Disability awareness involves self-reflection of one’s perceptions around disability and an active role in establishing inclusion for PWD.

The following 12 tips are provided for medical educators wishing to incorporate disability awareness into the medical training programs at their own institutions. These tips are based upon the authors’ experiences with disability awareness education in both undergraduate medical education (UME) and graduate medical education (GME) ( Parish *et al.*, 2024; Sinha *et al.*, 2024). These tips have been developed within the theoretical framework of intergroup contact theory (ICT), which stresses the importance of direct contact between minority and majority groups to break down prejudices and foster collaboration and shared understanding ( Pettigrew, 1998; Pettigrew & Tropp, 2006). A key tenet of any disability awareness program is the inclusion of PWD into every phase of the process, from development through implementation and assessment.

### Tip 1 – Embed persons with disabilities into all phases of the curriculum

Coined during the disability civil rights movement, the slogan “nothing about us without us” refers to the idea that no policy should be decided without involvement of members of the affected group ( Chu *et al.*, 2016). Multiple factors contribute to lower quality health care delivery to PWD, which contributes to further health care disparities and poorer health outcomes; most of these factors would be realistically modifiable with input from PWD ( Sharby *et al.*, 2015). PWD must be included in all aspects of disability awareness curricula, including the planning, development (design and content), implementation, and assessment phases. For the planning and development phases, we recommend including PWD in the brainstorming process and soliciting their input on most important needs and goals. For implementation, embed PWD into the curricular activities as experts and partner-educators. Students must have opportunities to directly interact with these individuals for success of the curriculum. Finally, during the assessment phase, solicit feedback from PWD and include their perceptions of the activities in the evaluation process. Holding a debrief session or focus group with these participants is a great way to obtain this key data.

Those who possess lived experiences and voices are most capable of making a longitudinal effect on future medical practice. Failure to engage with PWD can lead to the propagation of ableist ideas and distortion of the most important facets of disability and healthcare. Developing partnerships with local rehabilitation clinics and community centers are great ways to create professional contacts to foster a collaborative approach to disability awareness and recruit PWD to participate as educators.

### Tip 2 – Follow Kern’s six-step method for curriculum development

As with any curriculum development process, a thoughtfully constructed plan is essential to ensure success. We recommend Kern’s six-step model ( Thomas *et al.*, 2015). Step 1 is to conduct a general needs assessment for the medical school, and step 2 is to determine the specific needs of the medical students. Since every medical school is unique and medical students enter training from a wide array of backgrounds and experiences, there is no “one-size-fits-all" model for providing disability awareness education. A needs assessment survey and/or set of focus groups involving PWD, students, and other stakeholders can be used to tailor a disability awareness curriculum to meet the unique needs of each medical school. To ensure success, the recommendations of patients with disabilities in the local community should be solicited, to better understand what future physicians need to know. Although there are certainly recommendations that will be consistent from PWD across the globe, there may also be recommendations that are specific to a given community of which educators should be aware. For example, there may be specific cultural or institutional norms that medical students need to learn about for PWD in their community.

Learning objectives, step 3, should aim to address gaps in learner knowledge and align with programmatic learning goals. Published competencies such as those from the Alliance for Disability in Health Care Education can help guide the development of objectives ( ADHCE, 2024). When identifying educational strategies, step 4, be sure that they align with the overall curricular approach and culture of your medical school to establish continuity of instruction and further contribute to success. For example, if problem-based learning (PBL) is a core curricular element of your medical school, then including patients who have disabilities in one or more PBL cases would be an appropriate strategy. If students routinely engage in service learning, then partnering with community centers that provide services to PWD would be a great option. After step 5, implementation, a well-executed evaluation plan is pivotal to determine successes and areas of growth and improvement. As noted in Tip 1, be sure to include PWD into each phase of Kern’s model.

### Tip 3 – Utilize diverse clinicians with experience working with PWD

The Centers for Disease Control and Prevention classifies disability into six types: mobility, cognition, independent living, hearing, vision, and self-care ( CDC, 2024). Some medical and health professions, such as physical therapy, occupational therapy, speech-language pathology and physiatry, have specific roles in caring for PWD who have one or more of these disability types. Because of their prevalent roles working with this population, disability awareness is often an accreditation requirement and embedded within their training. For example, Doctor of Physical Therapy (DPT) programs integrate disability awareness, distinctively geared toward mobility-based disability, throughout their curricula ( Roush & Sharby, 2011). Nurses, who represent the largest workforce within healthcare, play diverse and often front-line roles in the care of PWD. Therefore, in addition to PWD, it is helpful to integrate healthcare professionals who have experience working with this population into the disability awareness programs for medical students. This is particularly important as PWD often require a team-based approach to care that includes a variety of health professions ( Reis *et al.*, 2004). Inclusion of clinicians who possess these experiences can help ensure that key considerations and clinical pearls are embedded within the curricula.

### Tip 4 – Include Interprofessional Education (IPE) elements

Emphasizing an interprofessional approach when implementing disability awareness curricula is important, because caring for PWD often requires communication and planning as a healthcare team ( Iezzoni, 2006; Reis *et al.*, 2004). IPE has been shown as beneficial to learning, skill development, and creating a plan of care ( McCave *et al.*, 2019; Visser *et al.*, 2019). It is also important for future physicians to understand the role of other healthcare professionals in treating PWD as it can allow for better patient-client management and referral for those who may have unmet needs. Since many medical schools have associated health professions programs, IPE activities can involve students from physical therapy, occupational therapy, speech-language pathology, nursing, and physician associate programs to name a few. Disability awareness activities provide a robust and practical setting to meet accreditation requirements around IPE.

### Tip 5 – Incorporate a broad spectrum of disability types into the curriculum

Disability is neither a single diagnosis nor a single human characteristic. It is a collection of physical or mental impairments that limits life activities, and it can result from myriad illnesses and injuries. Furthermore, people with disabilities are just as diverse as those who do not have a disability. Educational programs that promote disability awareness should strive to be as inclusive as possible and represent the full diversity of this population. One way to do this is to include a breadth of disability types in your instruction and learning experiences:

- Mobility – You could incorporate wheelchair users (manual and powerchair) and have students self-propel themselves in wheelchairs around your educational building.
- Cognition – You could allow students to volunteer at community centers or other sites that provide services to individuals with intellectual and developmental disabilities.
- Vision – You could incorporate a patient with a vision impairment into a PBL or case-based learning session or allow students to volunteer at training sites for service animals.
- Hearing – You could provide a medical sign language class or allow students to practice working with an interpreter to communicate with guests who have hearing impairments.

When adopting Tip 1, be sure to engage a diverse group of PWD in the planning and implementation of the program, ensuring diversity of age, sex, gender, race, ethnicity, disability type, disability etiology (congenital vs. acquired), occupation, etc.

### Tip 6 – Include a variety of pedagogies, emphasizing experiential learning

Understanding of disability awareness can be enhanced by using a multitude of pedagogies. While lectures, readings, videos or case studies might inform background information, such as the Americans with Disabilities Act (ADA) and communication etiquette, experiential learning provides hands-on experiences that can provide a unique method for learning. Experiential learning, including direct interaction between students and PWD, should be considered as a focal element of disability curricula. Furthermore, service learning and community outreach activities allow students to see and interact with PWD in non-clinical settings. Bringing PWD onto campus for small group discussions is another way to create valuable learning experiences. In the context of patients who may have intellectual and developmental disabilities or cognitive impairments, family members and caregivers can serve as valuable teachers in these experiential settings.

### Tip 7 – Ensure access to appropriate equipment and facilities

PWD often utilize assistive and adaptive equipment to help promote independence. As physicians are often the prescribers of equipment, it is imperative to have familiarity with types of equipment and spaces in which equipment is utilized. For example, a commonly prescribed piece of equipment for both short- and long-term mobility impairments is a wheelchair. Having various types of wheelchairs during interactive portions of disability awareness learning activities can allow students to use the equipment, which can spark discussion of costs, proper fit, and parts of wheelchairs that may be needed based on diagnosis. Similarly, gait belts and sliding boards are useful to have students practice how to transfer PWD on and off of an examination table. For language disabilities, communication devices should be used by students to experience how devices operate. Equipment integrated into the disability awareness curriculum should be diverse and based upon the type of disability being studied to allow for holistic exposure and understanding of the compatible devices that PWD may use.

### Tip 8 – Introduce students to the Americans with Disabilities Act (ADA)

The Americans with Disabilities Act (ADA) is a civil rights law that was enacted in 1990 to prohibit discrimination against individuals with disabilities, ensuring that this population has the same rights and opportunities as those without disabilities. Title III of the ADA sets the minimum standards for accessibility of public and privately-owned facilities, directs businesses to make reasonable accommodations when serving people with disabilities, and requires appropriate measures be taken to effectively communicate with individuals who have speech, hearing and vision impairments. Introducing medical students to the basic tenets of the ADA is an essential component of any disability awareness curriculum as it forms the foundation for government-mandated accommodation and accessibility considerations. Students should understand the legal implications of the ADA in their future clinical practices. This should begin as early as new student orientation when medical students are made aware of disability support services for students and processes for obtaining disability-related accommodations. As topics around disability in healthcare and considerations for patients who have specific types of disability are introduced in the formal curriculum, additional details regarding the ADA’s role in protecting the rights of PWD can be incorporated through short videos or reading assignments. The ADA National Network offers a variety of resources that could be used to serve this purpose ( ADATA, 2024). Other approaches that employ more active methods of instruction include one-on-one and small group conversations about accessibility with individuals who have disabilities and placing students into a variety of ADA compliant and non-compliant facilities. Longitudinal curricular elements such as clinical skills and learning communities offer opportunities for a threaded approach to education around the ADA. Our approach to teaching about the ADA includes both lecture-based content and experiential learning with early and longitudinal exposures.

### Tip 9 – Simulate first-hand experiences by placing learners “into the shoes” of PWD

It is important to note that although experiential learning may allow a student to simulate what it is like to have a disability, it is not the same as living with a disability. For our approach, PWD help to create and lead experiential learning activities. This allows medical students to have a more meaningful learning opportunity versus inducing a sympathy-based response for individuals who live with disabilities. For our curriculum, physical therapy students teach medical students how to self-propel a wheelchair. Medical students then self-propel wheelchairs through their educational building alongside individuals who use wheelchairs for mobility. The wheelchair users guide students to experience the ADA-compliant and non-compliant aspects of buildings, sidewalks, and parking during this process. The wheelchair users then have an open dialogue with students to discuss the long-term effects of using a wheelchair for mobility, share their experiences, and answer questions. These meaningful learning contexts can improve student engagement, understanding, and retention of the material all while promoting deeper awareness of the emotional, social, and physical aspects of living with a disability ( Laurillard, 2012). Immersing students in these situations helps break down stereotypes, while building a sense of responsibility to improve the lack of inclusivity, the “non-disability-friendly” spaces, and increase patience in their future clinical practice.

Educators should be aware that disability simulation can also have the unintended consequence of promoting ableism and discrimination against PWD. This can happen because in simulation students experience *becoming* disabled, thus overemphasizing trauma of the experience, which can promote pity, negative stereotypes, and underemphasize the social inequalities experienced by PWD over time ( Silverman, 2015). Caution must be taken when implementing any type of disability simulation, and it is paramount that PWD be involved in these simulations to mitigate these risks. Nonetheless, we feel that through thoughtful coaching and debriefing with PWD these unintended consequences can be avoided.

### Tip 10 – Create opportunities for open dialog between learners and PWD

Creating opportunities for open dialogue between students and PWD who serve as the facilitators of learning experiences is crucial to fostering a more effective learning environment. These interactions help dispel misconceptions, bridge gaps between learners and facilitators, and foster more encouraging and accepting clinical practice. PWD are underrepresented as educators, but they may reflect images in the mirror for many students and help guide learners in understanding everyday challenges and triumphs for PWD ( Anderson *et al.*, 1998). Open channels of communication in the learning environment help build collaborative relationships where facilitators and learners can discuss shared perspective and various views surrounding them as well as uncomfortable but necessary dilemmas ( Ta *et al.*, 2023). Whether in the form of a panel discussion or experiential learning activity in pre-clerkship training, a simulated patient encounter, or an authentic clinical experience during the third- and fourth-years, there are ample opportunities within the four-year medical curriculum to incorporate these discussions and effectively utilize PWD as educators. Including a minimum of one learning activity in the pre-clerkship phase and several authentic clinical encounters in the third- and fourth-years is recommended. Through open discussions, everyone’s unique abilities and perspectives are acknowledged and integrated to better enhance learning.

### Tip 11 – Embed reflective elements into the activities

Adding reflective elements, such as small group debriefs and reflection essays, into a disability curriculum can enhance an already dynamic and inclusive learning environment for learners and facilitators alike. For learners, research has shown that when students combine their learning with reflection, they are able to effectively understand and analyze problems that they could not otherwise ( Eyler & Giles, 1999). When students are encouraged to recognize their strengths and weaknesses and assess their learning through self-reflection, they are more likely to adopt those processes going forward ( Silverman & Cassaza, 2000). For facilitators, reflection provides structure to their teaching and encourages them to take responsibility for themselves. By understanding what is effective and what could be improved upon, reflection helps educators create more inclusive curricula with greater relevance and direction for their learners ( Daudelin, 1996). Reflective elements can easily be incorporated as part of experiential learning sessions (Tip 6) and open dialogue sessions (Tip 10). Ideally, reflection as a formal or informal learning element should accompany each component of a disability awareness curricular thread. Overall, incorporating reflective elements in a disability-based curriculum can enhance the experience for learners and stress continuity while also contributing to the professional growth of PWD as educators, creating a more refined ecosystem.

### Tip 12 – Create longitudinal experiences

Currently, only half of U.S. medical schools have a disability awareness program ( Seidel & Crowe, 2017). For those that do, most incorporate training within the pre-clinical years but fail to provide experiences in the clinical phase of training, which limits opportunities for application in clinical practice. Both the lack of education and continuity adds to the negative bias and discomfort that many medical students and physicians feel when treating PWD; this only results in worse healthcare and medical attention for PWD ( Sharby *et al.*, 2015). The ineffective nature of continued medical education contributes to the discrepancy between evidence/experience-based knowledge and fair practice ( Marinopoulos *et al.*, 2007). Thus, it is vital that schools not only incorporate disability training early in medical training but thread these experiences longitudinally throughout training. 

During the core clinical clerkships, ensure that students have the ability to interact with patients of *all* disability types. Internal medicine, pediatrics, and neurology clerkships, in particular, should offer ample opportunities for students to participate in the care of PWD from each of the six categories defined by the Centers for Disease Control and Prevention ( CDC, 2024). Thoughtful inclusion of disability awareness should also be incorporated into obstetrics and gynecology (OB/GYN) as well as pediatric clerkships in the context of parents who have a disability or parents who will be caring for children with a disability ( Amir *et al.*, 2022; Sheppard *et al.*, 2022). Asynchronous learning modules and simulated patient encounters can be used as supplements when students are unable to work with a specific type of disability. For example, embedding a patient who has a hearing or vision deficit or who uses a wheelchair into a simulation activity that already takes place within a clerkship is an effective way to add depth to a simulation without adding curricular time. Students should also participate in the development of management plans alongside physical or occupational therapists during their acting internships to strengthen their understanding of the complexities of caring for this patient population. Advanced electives in the care of PWD during the fourth year of medical school can provide further opportunities for students to deepen their skills prior to residency training.

## Conclusion

The goal of these 12 tips is to provide medical educators with the necessary tools to promote students’ awareness of unique considerations when providing care for PWD and foster a platform for PWD to function as key participants in the learning environment. It is crucial that medical schools prepare the next generation of physicians to provide effective health-care for this population through diverse teaching and learning methods, interprofessional collaboration, and a focus on reflection and continuity. By embracing these tips, the teaching and learning process can become enriching for all stakeholders and cultivate trainees who will be compassionate and confident providers within their future roles.

## Ethics and consent

Ethical approval and Consent were not required.

## Data Availability

No data are associated with this article.

## References

[ref-29] ADA National Network.2024; Retrieved 7 November 2024. https://adata.org/slo

[ref-30] ADHCE: Core competencies on disability for health care education.2024; Retrieved 7 November 2024. Reference Source

[ref-31] Amir N Smith LD Valentine AM : Clinician perspectives on the need for training on caring for pregnant women with intellectual and developmental disabilities. *Disabil Health J.* 2022;15(2): 101262. 10.1016/j.dhjo.2021.101262 35031269 PMC8983509

[ref-1] Anderson RJ Keller CE Karp JM : Enhancing diversity: educators with disabilities.Washington, D.C., Gallaudet University Press,1998. 10.2307/j.ctv2rcnggb

[ref-2] CDC: Disability and health overview.2024; Retrieved 18 March 2024. Reference Source

[ref-3] Chu LF Utengen A Kadry B : "Nothing about us without us"-patient partnership in medical conferences. *BMJ.* 2016;354: i3883. 10.1136/bmj.i3883 27628427

[ref-4] Daudelin MW : Learning from experience through reflection. *Organ Dyn.* 1996;24(3):36–48. 10.1016/S0090-2616(96)90004-2

[ref-5] Eyler J Giles D : Where's the learning in service learning.San Francisco, CA, Jossey-Bass Publishers,1999.

[ref-6] Holder M Waldman HB Hood H : Preparing health professionals to provide care to individuals with disabilities. *Int J Oral Sci.* 2009;1(2):66–71. 10.4248/ijos.09022 20687298 PMC3735794

[ref-7] Iezzoni LI : Going beyond disease to address disability. *N Engl J Med.* 2006;355(10):976–979. 10.1056/NEJMp068093 16957143

[ref-8] Laurillard D : Teaching as a design science: building pedagogical patterns for learning and technology.London, U.K., Routledge,2012. Reference Source

[ref-9] Leppert R Schaeffer K : 8 facts about Americans with disabilities. 2023; Retrieved 18 March 2024. Reference Source

[ref-10] Marinopoulos SS Dorman T Ratanawongsa N : Effectiveness of continuing medical education. *Evid Rep Technol Assess (Full Rep).* 2007; (149):1–69. 17764217 PMC4781050

[ref-11] McCave EL Aptaker D Hartmann KD : Promoting affirmative transgender health care practice within hospitals: an IPE standardized patient simulation for graduate health care learners. *MedEdPORTAL.* 2019;15: 10861. 10.15766/mep_2374-8265.10861 32051844 PMC7010321

[ref-12] McMillan C Lee J Milligan J : Physician perspectives on care of individuals with severe mobility impairments in primary care in Southwestern Ontario, Canada. *Health Soc Care Community.* 2016;24(4):463–472. 10.1111/hsc.12228 25809600

[ref-13] Okoro CA Hollis ND Cyrus AC : Prevalence of disabilities and health care access by disability status and type among adults - United States, 2016. *MMWR Morb Mortal Wkly Rep.* 2018;67(32):882–887. 10.15585/mmwr.mm6732a3 30114005 PMC6095650

[ref-14] Parish A Carver C Lein DH Jr : Come Roll with Me: an interprofessional experience to promote disability awareness. *Teach Learn Med.* 2024;36(2):183–197. 10.1080/10401334.2022.2148107 36426664

[ref-15] Pettigrew TF : Intergroup contact theory. *Annu Rev Psychol.* 1998;49:65–85. 10.1146/annurev.psych.49.1.65 15012467

[ref-16] Pettigrew TF Tropp LR : A meta-analytic test of intergroup contact theory. *J Pers Soc Psychol.* 2006;90(5):751–783. 10.1037/0022-3514.90.5.751 16737372

[ref-17] Reis JP Breslin ML Iezzoni LI : It takes more than ramps to solve the crisis of healthcare for people with disabilities.Chicago, IL, Rehabilitation Institute of Chicago,2004. Reference Source

[ref-18] Roush SE Sharby N : Disability reconsidered: the paradox of physical therapy. *Phys Ther.* 2011;91(12):1715–1727. 10.2522/ptj.20100389 22003161

[ref-24] Seidel E Crowe S : The state of disability awareness in American medical schools. *Am J Phys Med Rehabil.* 2017;96(9):673–676. 10.1097/PHM.0000000000000719 28187005

[ref-19] Sharby N Martire K Iversen MD : Decreasing health disparities for people with disabilities through improved communication strategies and awareness. *Int J Environ Res Public Health.* 2015;12(3):3301–3316. 10.3390/ijerph120303301 25809511 PMC4377965

[ref-27] Sheppard ME Vitalone-Raccaro N Kaari JM : Planting the seeds of collaboration: a pilot for school/clinic partnerships during pediatric clerkship. *Intellect Dev Disabil.* 2022;60(6):453–464. 10.1352/1934-9556-60.6.453 36454613

[ref-25] Silverman AM : The perils of playing blind: problems with blindness simulation, and a better way to teach about blindness. *J of Blindness Innovation and Res.* 2015;5(2). 10.5241/5-81

[ref-20] Silverman S Cassaza M : Learning and development: making connections to enhance teaching.San Francisco, CA, Jossey-Bass Publishers,2000. Reference Source

[ref-21] Sinha T Parish A Lein DH : Integration of disability awareness improves medical students’ attitudes toward people with disabilities. *Med Sci Educ.* 2024;34(3):561–569. 10.1007/s40670-024-02004-0 38887426 PMC11180063

[ref-22] Ta T Geron T Levinson M : "The power of open dialogue": using normative case studies to facilitate ethical dilemmas discussions among school teachers. *Teaching and Teacher Education.* 2023;132: 104237. 10.1016/j.tate.2023.104237

[ref-26] Thomas PA Kern DE Hughes MT : Curriculum Development for Medical Education: a six-step approach. Johns Hopkins University Press,2015. Reference Source

[ref-23] Visser CLF Kusurkar RA Croiset G : Students' motivation for interprofessional collaboration after their experience on an IPE ward: a qualitative analysis framed by self-determination theory. *Med Teach.* 2019;41(1):44–52. 10.1080/0142159X.2018.1436759 29490575

