# Supplementary material for: 12 Tips for Including Disability Awareness within Undergraduate Medical Education Curricula
Source: MedEdPublish (2016). 2024 Dec 10;14:32. Originally published 2024 May 14. [Version 3] doi: 10.12688/mep.20329.3 (PMC11574333; doi:10.12688/mep.20329.3)
Supplement: Peer review history

# Reviewer report: Reviewer response for version 3

**DOI:** 10.21956/mep.22309.r40746

**Published:** 2025-01-13

**Authors:** Daniëlle Verstegen

**Affiliations:** 1 School of Health Professions Education, Maastricht University, Maastricht, The Netherlands

Thank you for the opportunity to review the article '12 Tips for Including Disability Awareness within Undergraduate Medical Education Curricula'.

 The article contains a wealth of experience, insights and tips on how to include disability awareness in undergraduate curricula. There is some overlap between the tips, but that does not disturb me. It is very US-centered, and that makes some of the tips less useful for readers based elsewhere. It would be easy to partly address this, for example by changing Tip 8 to 'Introducing students to legal frameworks' instead of specifically the American Disability Act. Or by acknowledging that the classification of disabilities or even the definition could be different elsewhere (e.g. I am surprised not to see categories related to mental health and (health) literacy).

 I am an Instructional Designer by background. So, I fully agree that a systematic approach to curriculum design is a good tip. And I agree that Kern's model is suitable, but it is not the only good model. So, I would rather change Tip 2 to 'Take a systematic approach to curriculum development' and use Kern's model as an example of a good model.

 That same background makes me curious for more information about Tip 6: include a variety of pedagogical approaches. Could you possibly elaborate a bit more?

 I would also say that Tip 11 is a vital tip to go with Tip 6. I would place them together.

 Finally, two more practical things:

- Especially in the abstract it is not very clear whether 'working with persons with disabilities refers to working with patients with disabilities or to working with colleagues or students with disabilities.
- And would it be possible to leverage the experiences of students and staff with disabilities (or with family members with disabilities) to strengthen the disability curriculum? My experience as a PBL tutor and teacher is that this can be very powerful.

If evidence from practice is presented, are all the underlying source data available to ensure full reproducibility?

Not applicable

Is the topic of the practical tips discussed accurately in the context of the current literature

Yes

Are all factual statements correct and adequately supported by citations?

Yes

Are the conclusions drawn balanced and justified on the basis of the presented arguments?

Yes

Are arguments sufficiently supported by evidence from the published literature and/or the authors’ practice?

Yes

Reviewer Expertise:

I am an instructional designer with much experience in curriculum design. I am not a physician and not an expert on disability curriculum

I confirm that I have read this submission and believe that I have an appropriate level of expertise to confirm that it is of an acceptable scientific standard.

# Reviewer report: Reviewer response for version 3

**DOI:** 10.21956/mep.22309.r40510

**Published:** 2025-01-10

**Authors:** Natalie L. Shaheen

**Affiliations:** 1 Illinois State University, Normal, Illinois, USA

I appreciate and support the broad aim and argument of this paper. Healthcare professionals need more knowledge about disabled people in order to provide culturally responsive care.

 This paper has three primary weaknesses that must be addressed.

1. Simulations: I acknowledge that the authors have attempted to address the concerns a previous reviewer offered about the discussion of disability simulations. However, the additions do not go far enough. They briefly nod at the literature that says disability simulations are problematic. But their framing, and minimal citation of the literature, makes it seem like the reinforcement of misconceptions is an uncommon side effect of disability simulations, which is not what the literature says. The literature on this topic is fairly robust. Silverman’s 2015 ^3^ piece is a useful citation. But I’d encourage the authors to read deeper. The authors should also offer an alternative to disability simulations for others to consider. For example, instead of a wheelchair simulation, students could use the ADA regulations to do an audit of the campus environment on their own. Then they could conduct a second audit of the same space in collaboration with wheelchair users and realize how many barriers they missed.
2. Implicit Bias: An examination of students’ implicit bias should be part of a curriculum that aims to improve preservice healthcare professionals work with disabled people. This could easily be incorporated into tip 11, which pertains to reflection. For example, Module 5 from the OER *Disability, Learning, and Education: A Guidebook* demonstrates how implicit bias is explored in an undergraduate education course. Additionally, Chapter 4 out of the short book *Demystifying Disability* (Ladau, 2021 ^2^) is helpful in explorations of implicit bias.
3. Accessible Collaboration: The authors discuss the importance of engaging disabled people in curriculum development work, I applaud them for that emphasis. However, they neglect to point out that the standard collaboration processes in academia are often inaccessible to disabled people. If medical school faculty are going to invite disabled people to collaborate in curriculum development, they must ensure that their ways of working are accessible to disabled people, otherwise they risk causing harm.

Other, less critical, weaknesses that I would encourage the authors to consider addressing are:

1. The authors did not indicate what role, if any, disabled people had in conceptualizing and writing the article.
2. When discussing developing partnerships, I would suggest the authors add organizations of disabled people to their list of suggestions. Rehabilitation clinics and community centers are often run by nondisabled people and have a different worldview than organizations of disabled people. Organizations of disabled people that the authors could suggest including Autistic Self Advocacy Network, National Association of the Deaf, and National Federation of the Blind.
3. All discussion of disabled people in this article presupposes that disabled people are always patients in medical settings. But disabled people are also doctors, nurses, social workers, psychologists, etc. For that matter, there are disabled students in undergraduate medical programs. I encourage the authors to consider incorporating this reality into their tips. For example, how could disabled medical students help teach their peers about disability? How could a local disabled physician collaborate with the medical school in developing a disability awareness curriculum?

If evidence from practice is presented, are all the underlying source data available to ensure full reproducibility?

Not applicable

Is the topic of the practical tips discussed accurately in the context of the current literature

Partly

Are all factual statements correct and adequately supported by citations?

Yes

Are the conclusions drawn balanced and justified on the basis of the presented arguments?

Partly

Are arguments sufficiently supported by evidence from the published literature and/or the authors’ practice?

Partly

Reviewer Expertise:

Digital accessibility, disability studies, K-12 education, blindness, accessible STEM education

I confirm that I have read this submission and believe that I have an appropriate level of expertise to state that I do not consider it to be of an acceptable scientific standard, for reasons outlined above.

## References

[1] : Disability, Learning, and Education: A Guidebook.2024; 10.30707/1728998113.847324 10.30707/1728998113.847324

[2] : Demystifying Disability What to Know, What to Say, and How to Be an Ally.2021; Reference source

[3] : The Perils of Playing Blind: Problems with Blindness Simulation and a Better Way to Teach about Blindness. *Journal of Blindness Innovation and Research* .2015;5(2) : 10.5241/5-81 10.5241/5-81

# Reviewer report: Reviewer response for version 3

**DOI:** 10.21956/mep.22309.r40496

**Published:** 2024-12-20

**Authors:** Suzanne Smeltzer

**Affiliations:** 1 Villanova University, Pennsylvania, USA

I have read this submission. I believe that I have an appropriate level of expertise to confirm that it is of an acceptable scientific standard. Further, the information contained in the manuscript is important and should be useful to individuals and programs that are planning strategies to address disability in medical school curricula (as well as curricula of other healthcare professions programs).

If evidence from practice is presented, are all the underlying source data available to ensure full reproducibility?

Yes

Is the topic of the practical tips discussed accurately in the context of the current literature

Yes

Are all factual statements correct and adequately supported by citations?

Partly

Are the conclusions drawn balanced and justified on the basis of the presented arguments?

Partly

Are arguments sufficiently supported by evidence from the published literature and/or the authors’ practice?

Partly

Reviewer Expertise:

NA

I confirm that I have read this submission and believe that I have an appropriate level of expertise to confirm that it is of an acceptable scientific standard.

# Reviewer report: Reviewer response for version 2

**DOI:** 10.21956/mep.22106.r38860

**Published:** 2024-11-18

**Authors:** Linda S Nield

**Affiliations:** 1 West Virginia University School of Medicine, Morgantown, West Virginia, USA

Thank you for the opportunity to review the revised work of Sinha and colleagues. Their twelve tips are comprehensive and clear and will help enhance medical education on this topic. The one minor edit I recommend is to add “(PWD)” to the end of the first sentence of the Abstract to explain this abbreviation which is subsequently used again in the Abstract.

 Approval status is "Approved" with the one suggested minor edit as mentioned in my review.

If evidence from practice is presented, are all the underlying source data available to ensure full reproducibility?

Partly

Is the topic of the practical tips discussed accurately in the context of the current literature

Yes

Are all factual statements correct and adequately supported by citations?

Partly

Are the conclusions drawn balanced and justified on the basis of the presented arguments?

Partly

Are arguments sufficiently supported by evidence from the published literature and/or the authors’ practice?

Partly

Reviewer Expertise:

medical education; MD admissions

I confirm that I have read this submission and believe that I have an appropriate level of expertise to confirm that it is of an acceptable scientific standard.

# Reviewer report: Reviewer response for version 2

**DOI:** 10.21956/mep.22106.r39201

**Published:** 2024-10-24

**Authors:** Suzanne Smeltzer

**Affiliations:** 1 Villanova University, Pennsylvania, USA

While the manuscript addresses an important topic, that is, the need to address disability in medical education, there are some issues that bear authors’ attention:

1. The estimate of prevalence of disability is low. In 2024, the CDC reported 70 million people in USA to have a disability.
2. Document refers to “medical” care; reviewer would suggest use of “health” care as it is more encompassing and does include medical care.
3. Authors state that “Approximately half of American medical schools have a disability curriculum”, but say nothing about the quality of those curricula. It seems important to do so as not all curricula are the same.
4. Tip 1: would change “ these factors would be realistically modifiable” to “ most of these factors”.
5. Some elaboration of how the steps of Kern’s approach (mentioned at end of Tip 2) would be accomplished would be helpful addition to Tip 1. 
6. Tip 3 makes no mention of the largest workforce in health care (nursing workforce); so it should be mentioned when other healthcare professions are mentioned.  Nursing IS mentioned in Tip 4 (once, but discussion is cursory).
7. Tip 6 does not mention family members/caregivers who might be the best informants, especially of individuals with significant IDD or cognitive impairment; so they should be mentioned as possible informants.
8. Tip 8 should mention that many resources…videos, etc….can be accessed for education of students. Inclusion of mention of some examples of these resources would likely be helpful.
9. Tip 9: Should emphasize that the ADA has legal implications; otherwise, too many health care professionals think of ADA requirements as “nice to know” rather than a legal mandate.
10. While the caution about disability simulation experiences was an extremely important addition, another possible negative consequence of these experiences is that “learners” often approach them as a game or a joke. Instead of concluding that having a disability is worse than death (which is the view of too many HCPs), some come away from the experience with the view that it is “not a big deal”. So great caution in using such simulations should be emphasized and PWD must be involved in these simulations. Otherwise, they should be avoided.
11. Tip 12 does not address OB/maternal care and it must do so as women with disability face ableism to a high degree because HCPs fail to consider them appropriate parents (includes males with disability, too). So it must be mentioned as an area of the curriculum and practice where disability is addressed.
12. An issue not addressed in the 12 tips is what content about disability should be addressed and what competencies should be the goal of medical education. I would recommend that the authors review the Core Competencies developed by the Alliance for Disability in Health Care Education, Inc. (ADHCE).  The competencies were developed by an interprofessional, multidisciplinary group of healthcare professionals and individuals with disability. The competencies have been reviewed and endorsed by disability and disability advocacy groups, and major professional organizations, including those from medicine. The National Council on Disability (2022) also endorsed the competencies for use in the education of health care professionals across disciplines. The content of what is or should be taught is missing from the manuscript.
13. A point worth considering for inclusion is that faculty (and many physicians currently in practice) may have received little if any education about the topic in their own education and training. Thus, they, too, will need to receive help in identifying 1) why this important, and 2) how having a disability affects one’s ability to receive health care. Further, HCP faculty often believe that they are already teaching adequately about disability (if they are reaching about disabling conditions, which is not the same as teaching about disability), so will be reluctant to add or take on another topic. This needs to be considered in the process and the importance of addressing disability emphasized.

If evidence from practice is presented, are all the underlying source data available to ensure full reproducibility?

Yes

Is the topic of the practical tips discussed accurately in the context of the current literature

Yes

Are all factual statements correct and adequately supported by citations?

Partly

Are the conclusions drawn balanced and justified on the basis of the presented arguments?

Partly

Are arguments sufficiently supported by evidence from the published literature and/or the authors’ practice?

Partly

Reviewer Expertise:

NA

I confirm that I have read this submission and believe that I have an appropriate level of expertise to confirm that it is of an acceptable scientific standard, however I have significant reservations, as outlined above.

# Response

**Published:** 2024-12-05

**Authors:** Will Brooks

**Affiliations:** The University of Alabama at Birmingham, Birmingham, Alabama, USA

1. The estimate of prevalence of disability is low. In 2024, the CDC reported 70 million people in USA to have a disability. Thank you for this updated statistic. We have edited the manuscript to reflect the current estimate of 70 million.
2.  Document refers to “medical” care; reviewer would suggest use of “health” care as it is more encompassing and does include medical care. We have changed all instances of “medical care” with “healthcare”.
3.  Authors state that “Approximately half of American medical schools have a disability curriculum” but say nothing about the quality of those curricula. It seems important to do so as not all curricula are the same. We agree with the reviewer. Unfortunately, we are not aware of any data that would speak to the quality of medical school disability curricula. Quality likely varies and would be an interesting point of study for future work in this area.  
4. Tip 1: would change “these factors would be realistically modifiable” to “most of these factors”. We have made this adjustment.  
5. Some elaboration of how the steps of Kern’s approach (mentioned at end of Tip 2) would be accomplished would be helpful addition to Tip 1. We have added additional suggestions into Tip 1 for how to utilize PWD effectively into each general phase of the curriculum, including design, implementation, and assessment. For educators who are looking for more detailed ideas regarding implementation of Kern’s approach, his book, Curriculum Development for Medical Education, would be an appropriate source.  
6. Tip 3 makes no mention of the largest workforce in health care (nursing workforce); so, it should be mentioned when other healthcare professions are mentioned.  Nursing IS mentioned in Tip 4 (once, but discussion is cursory). Thank you for pointing out this important omission. We have added the following statement to Tip 3: “Nurses, who represent the largest workforce within healthcare, play diverse and often front-line roles in the care of PWD.”
7. Tip 6 does not mention family members/caregivers who might be the best informants, especially of individuals with significant IDD or cognitive impairment; so, they should be mentioned as possible informants. This is a great point. We have added the following sentence to Tip 6: “In the context of patients who may have intellectual and developmental disabilities or cognitive impairments, family members and caregivers can serve as valuable teachers in these experiential settings.”  
8. Tip 8 should mention that many resources…videos, etc…. can be accessed for education of students. Inclusion of mention of some examples of these resources would likely be helpful. Thank you. We have added a reference to the ADA National Network, which provides a variety of resources on understanding the ADA.  
9. Tip 9: Should emphasize that the ADA has legal implications; otherwise, too many health care professionals think of ADA requirements as “nice to know” rather than a legal mandate. We have added a statement to Tip 8 on the importance of students understanding the legal implications of the ADA in their future clinical practices.  
10. While the caution about disability simulation experiences was an extremely important addition, another possible negative consequence of these experiences is that “learners” often approach them as a game or a joke. Instead of concluding that having a disability is worse than death (which is the view of too many HCPs), some come away from the experience with the view that it is “not a big deal”. So great caution in using such simulations should be emphasized and PWD must be involved in these simulations. Otherwise, they should be avoided. We agree with the reviewer and have added another statement to Tip 9 emphasizing caution and the inclusion of PWD in any disability simulation to mitigate risks.  
11. Tip 12 does not address OB/maternal care, and it must do so as women with disability face ableism to a high degree because HCPs fail to consider them appropriate parents (includes males with disability, too). So it must be mentioned as an area of the curriculum and practice where disability is addressed. This is another great suggestion. We have added a statement to Tip 12 calling for “Thoughtful inclusion of disability awareness should also be incorporated into obstetrics and gynecology (OB/GYN) as well as pediatric clerkships in the context of parents who have a disability or parents who will be caring for children with a disability”. Two additional citations were added to support this.  
12. An issue not addressed in the 12 tips is what content about disability should be addressed and what competencies should be the goal of medical education. I would recommend that the authors review the Core Competencies developed by the Alliance for Disability in Health Care Education, Inc. (ADHCE).  The competencies were developed by an interprofessional, multidisciplinary group of healthcare professionals and individuals with disability. The competencies have been reviewed and endorsed by disability and disability advocacy groups, and major professional organizations, including those from medicine. The National Council on Disability (2022) also endorsed the competencies for use in the education of health care professionals across disciplines. The content of what is or should be taught is missing from the manuscript. While we feel that specifying the exact content and competencies for UME disability education is outside of the scope of this particular 12 tips article and will likely vary between institutions, your point of providing recommended guidance on published competencies is well taken. To that end, we have included the ADHCE as an example of competencies for medical educators to consider when developing their own learning objectives in Tip 2.  
13. A point worth considering for inclusion is that faculty (and many physicians currently in practice) may have received little if any education about the topic in their own education and training. Thus, they, too, will need to receive help in identifying 1) why this important, and 2) how having a disability affects one’s ability to receive health care. Further, HCP faculty often believe that they are already teaching adequately about disability (if they are reaching about disabling conditions, which is not the same as teaching about disability), so will be reluctant to add or take on another topic. This needs to be considered in the process and the importance of addressing disability emphasized. This is an important point and one that we agree should be included in the manuscript. We have added the following to the Introduction: “It must be noted that instruction regarding the pathophysiology, diagnosis and management of disabling conditions is not equivalent to instruction regarding disability. Disability awareness involves self-reflection of one’s perceptions around disability and an active role in establishing inclusion for PWD.”

# Reviewer report: Reviewer response for version 1

**DOI:** 10.21956/mep.21759.r37359

**Published:** 2024-06-19

**Authors:** Linda S Nield

**Affiliations:** 1 West Virginia University School of Medicine, Morgantown, West Virginia, USA

Thank you for the opportunity to review the "12 Tips for Including Disability Awareness within UGME Curricula."  The topic is important and an area that must be enhanced in medical school curricula. The authors provide a valuable approach to increasing the topic throughout the curriculum; however, each individual tip could be abbreviated and more focused on one issue; with the provision of at least one concrete example of a learning event included in each tip.

 Suggested edits to this work are as follows:

 Abstract:  The first two sentences include information which requires citations.  Typically, citations must not be included in an abstract as the abstract must be able to stand alone. Perhaps re-wording is needed here so citations are not required.

 Tip 1: There are multiple tips included in this one tip.  Perhaps this first tip should be limited to the concept of "identifying gaps."

 Tip 2:  No recommended edits

 Tip 3:  No recommended edits

 Tip 4:  No recommended edits

 Tip 5: Comment-This tip is quite similar to Tip 2, regarding the need to include persons with disabilities in the process of creating and embedding learning events throughout the curriculum.  

 Tip 6:  The first sentence is somewhat awkward, therefore, can it be reworded?

 Tip 7:  Can the authors provide an example of how equipment could be included in an actual learning event?  As is written now ("Having access to various types of wheelchairs during interactive...") it is unclear exactly how to include the equipment in teaching.

 Tip 8: Can authors provide more guidance and be more specific about where, when and how often the ADA should be incorporated into the curriculum?  

 Tip 9:  Main reason for the "partly" rating. The authors must consider adding information about any potential negatives to "placing learners into the shoes" of persons with disabilities, such as discussed in the work of Dr. Arielle Michal Silverman.  See citation from 2015. (Ref -1)

 Tip 10: Authors should provide more detail about where, when and how often to include these open dialogues in the curriculum, and in what format.

 Tip 11:  please see critique of Tip 10.

 Tip 12:  please see critique of Tip 10. The last sentence about residency programs should be deleted, as these tips are for UGME only.

If evidence from practice is presented, are all the underlying source data available to ensure full reproducibility?

Partly

Is the topic of the practical tips discussed accurately in the context of the current literature

Yes

Are all factual statements correct and adequately supported by citations?

Partly

Are the conclusions drawn balanced and justified on the basis of the presented arguments?

Partly

Are arguments sufficiently supported by evidence from the published literature and/or the authors’ practice?

Partly

Reviewer Expertise:

medical education; MD admissions

I confirm that I have read this submission and believe that I have an appropriate level of expertise to confirm that it is of an acceptable scientific standard, however I have significant reservations, as outlined above.

## References

[1] : The Perils of Playing Blind: Problems with Blindness Simulation and a Better Way to Teach about Blindness. *Journal of Blindness Innovation and Research* .2015;5(2) : 10.5241/5-81 10.5241/5-81

# Response

**Published:** 2024-09-04

**Authors:** Will Brooks

**Affiliations:** The University of Alabama at Birmingham, Birmingham, Alabama, USA

Thank you for the helpful review of our manuscript. Below is a point-by-point description of revisions based upon your feedback.

**Abstract:** 

**Reviewer Comment: **The first two sentences include information which requires citations.  Typically, citations must not be included in an abstract as the abstract must be able to stand alone.

**Response:** Perhaps re-wording is needed here so citations are not required. These sentences were re-worded.

**Reviewer Comment: **Tip 1: There are multiple tips included in this one tip.  Perhaps this first tip should be limited to the concept of "identifying gaps."

**Response:** We have edited the former Tip 1 (now Tip 2 in the revised manuscript) as “Follow Kern’s six-step method for curriculum development”. While this remains a multi-part process, the overall tip is now condensed into one coherent and well-described process.  

**Reviewer Comment: **Tip 2:  No recommended edits Tip 3:  No recommended edits Tip 4:  No recommended edits

**Reviewer Comment: **Tip 5: Comment-This tip is quite similar to Tip 2, regarding the need to include persons with disabilities in the process of creating and embedding learning events throughout the curriculum. 

**Response:** ​​​​​​​This tip was edited to clarify the emphasis on the need to include a broad diversity of disability types in curricula.

**Reviewer Comment: **Tip 6:  The first sentence is somewhat awkward, therefore, can it be reworded?

**Response:** ​​​​​​​The first sentence was rewritten and integrated to make the paragraph sound more concise.

**Reviewer Comment: **Tip 7:  Can the authors provide an example of how equipment could be included in an actual learning event? 

**Response:** ​​​​​​​As is written now ("Having access to various types of wheelchairs during interactive...") it is unclear exactly how to include the equipment in teaching. More information was added to demonstrate how equipment can be used versus just having access to it.

**Reviewer Comment: **Tip 8: Can authors provide more guidance and be more specific about where, when and how often the ADA should be incorporated into the curriculum? 

**Response:** ​​​​​​​Further information has been included in this Tip regarding how the ADA could be incorporated in the curriculum. We have recommended early exposure to coincide with school or course orientation, when information for students who have disabilities is made available and then longitudinal education as part of clinical skills or learning communities programs.

**Reviewer Comment: **Tip 9:  Main reason for the "partly" rating. The authors must consider adding information about any potential negatives to "placing learners into the shoes" of persons with disabilities, such as discussed in the work of Dr. Arielle Michal Silverman.  See citation from 2015. (Ref -1)

**Response:** ​​​​​​​Thanks for this helpful suggestion.  This information has been added.

**Reviewer Comment: **Tip 10: Authors should provide more detail about where, when and how often to include these open dialogues in the curriculum, and in what format.

**Response:** ​​​​​​​Thank you. While there is no prescription for necessary timing of open dialogue, we have provided a list of options for successful incorporation during the pre-clinical and clinical years of training. We have recommended at least 1 activity in the preclinical phase and several authentic clinical encounters in the third- and fourth-years.

**Reviewer Comment: **Tip 11:  please see critique of Tip 10.

**Response:** We have added the following to Tip 11: “Reflective elements can easily be incorporated as part of experiential learning sessions (Tip 9) and open dialogue sessions (Tip 10).  Ideally, reflection as a formal or informal learning element should accompany each component of a disability awareness curricular thread.

**Reviewer Comment: **Tip 12:  please see critique of Tip 10. The last sentence about residency programs should be deleted, as these tips are for UGME only.

**Response:** ​​​​​​​We have edited Tip 12 to include more specific recommendations around clerkships, acting internships, and advanced clinical electives. We have also removed the last sentence about residency programs as suggested.

# Reviewer report: Reviewer response for version 1

**DOI:** 10.21956/mep.21759.r37148

**Published:** 2024-06-13

**Authors:** Ami L DeWaters

**Affiliations:** 1 Penn State College of Medicine, Hershey, Pennsylvania, USA

This 12 tips article summarizes recommendations for building curricula within undergraduate medical education on the topic of disability awareness. 

 While this is undoubtedly an extremely important topic, and the authors are to be commended for tackling it, the tips are currently too general to provide insight into the unique considerations for disability content. 

 In particular, tips 1, 6, 11, and 12, are tips that would apply to any curricular innovation, and is not specific to disability. Therefore, I would recommend either replacing these with more specific and relevant tips to disability curricula, or condensing them into one tip, such as "follow Kern's method for curriculum development."

 In addition, and most importantly, one of the most important aspects of developing curriculum on caring for patients with disabilities is not listed as a tip; it is essential that persons with disabilities be included in the design of the curriculum. In my opinion, this must be included as a tip in any perspective piece about building curricula on disability awareness.

If evidence from practice is presented, are all the underlying source data available to ensure full reproducibility?

Yes

Is the topic of the practical tips discussed accurately in the context of the current literature

Partly

Are all factual statements correct and adequately supported by citations?

Yes

Are the conclusions drawn balanced and justified on the basis of the presented arguments?

Partly

Are arguments sufficiently supported by evidence from the published literature and/or the authors’ practice?

Partly

Reviewer Expertise:

Curriculum development, health systems science, systems-based practice, disability education

I confirm that I have read this submission and believe that I have an appropriate level of expertise to confirm that it is of an acceptable scientific standard, however I have significant reservations, as outlined above.

# Response

**Published:** 2024-09-04

**Authors:** Will Brooks

**Affiliations:** The University of Alabama at Birmingham, Birmingham, Alabama, USA

Thank you for the helpful review of our manuscript. 

 We have re-titled the former Tip 1 (now Tip 2 in the revised manuscript) as “Follow Kern’s six-step method for curriculum development” and reworked this section to include each of the six phases described in his book. We do not feel that Tips 6 (Include a variety of pedagogies, emphasizing experiential learning), 11 (Embed reflective elements), and 12 (Create longitudinal experiences) fit precisely within Kern’s model, yet are important in the context of disability awareness promotion.

 Therefore, we have elected to keep those as separate and unique tips to highlight their importance. The title and focus of the former Tip 2 is “Embed PWD into all phases of the curriculum”.

 We agree wholeheartedly with the reviewer that persons with disabilities MUST be included in the planning, design, implementation, and evaluation of a disability awareness curriculum. We have elevated this to Tip 1 in the revised manuscript and replaced the abbreviation “PWD” from the title with “persons with disabilities” to accentuate this tip and highlight its importance.
